# Consistent Cerebral Blood Flow Covariance Networks across Healthy Individuals and Their Similarity with Resting State Networks and Vascular Territories

**DOI:** 10.3390/diagnostics10110963

**Published:** 2020-11-17

**Authors:** Alice Pirastru, Laura Pelizzari, Niels Bergsland, Marta Cazzoli, Pietro Cecconi, Francesca Baglio, Maria Marcella Laganà

**Affiliations:** 1 IRCCS, Fondazione Don Carlo Gnocchi ONLUS, 20148 Milan, Italy; apirastru@dongnocchi.it (A.P.); lapelizzari@dongnocchi.it (L.P.); nbergsland@dongnocchi.it (N.B.); mcazzoli@dongnocchi.it (M.C.); pcecconi@dongnocchi.it (P.C.); mlagana@dongnocchi.it (M.M.L.); 2 Buffalo Neuroimaging Analysis Center, Department of Neurology, School of Medicine and Biomedical Sciences, University at Buffalo, State University of New York, Buffalo, NY 14203, USA

**Keywords:** MRI, functional MRI, cerebral blood flow, arterial spin labeling, resting state networks, vascular territories, independent component analysis

## Abstract

Cerebral blood flow (CBF) represents the local blood supply to the brain, and it can be considered a proxy for neuronal activation. Independent component analysis (ICA) can be applied to CBF maps to derive patterns of spatial covariance across subjects. In the present study, we aimed to assess the consistency of the independent components derived from CBF maps (CBF-ICs) across a cohort of 92 healthy individuals. Moreover, we evaluated the spatial similarity of CBF-ICs with respect to resting state networks (RSNs) and vascular territories (VTs). The data were acquired on a 1.5 T scanner using arterial spin labeling (ASL) and resting state functional magnetic resonance imaging. Similarity was assessed considering the entire ASL dataset. Consistency was evaluated by splitting the dataset into subsamples according to three different criteria: (1) random split of age and sex-matched subjects, (2) elderly vs. young, and (3) males vs. females. After standard preprocessing, ICA was performed. Both consistency and similarity were assessed by visually comparing the CBF-ICs. Then, the degree of spatial overlap was quantified with Dice Similarity Coefficient (DSC). Frontal, left, and right occipital, cerebellar, and thalamic CBF-ICs were consistently identified among the subsamples, independently of age and sex, with fair to moderate overlap (0.2 < DSC ≤ 0.6). These regions are functional hubs, and their involvement in many neurodegenerative pathologies has been observed. As slight to moderate overlap (0.2< DSC < 0.5) was observed between CBF-ICs and some RSNs and VTs, CBF-ICs may mirror a combination of both functional and vascular brain properties.

## 1. Introduction

Arterial spin labeling (ASL) is a widely used noninvasive magnetic resonance imaging (MRI) technique which provides quantitative and biologically meaningful maps of cerebral blood flow (CBF) by using blood as an endogenous contrast agent. CBF maps represent the blood volume flowing in the brain tissue in a given time per unit mass. Therefore, CBF is closely linked to cerebral metabolism and can be considered a surrogate measure of brain activity; it also indirectly reflects brain vascular health. For these reasons, CBF has been investigated both in physiological and pathological conditions. Changes in perfusion have been detected in normal aging, independently of gray matter (GM) atrophy [1]. Furthermore, CBF alterations have been reported in presence of some neurodegenerative diseases such as Parkinson’s disease (PD) [2,3], Alzheimer’s disease (AD) [4,5], frontotemporal dementia [5,6], and multiple sclerosis (MS) [7,8]. Associations between brain perfusion and either disability or cognitive impairment were also observed even in the early disease stages of PD [9,10], as well as in subjects with MS [11,12] and AD [13]. Thus, measurement of CBF may be capable of capturing either early metabolic/functional or vascular changes underlying neurodegeneration. Having a noninvasive understanding of if differential patterns of perfusion alterations can be associated with specific neurological diseases is of great interest.

Independent component analysis (ICA) is a signal processing approach that can be used to process MRI data, leading to the identification of patterns of temporal and/or spatial covariance, named independent components (ICs) [14]. This technique has been widely used with resting state functional MRI (rsfMRI) [15], to derive the well-established resting state networks (RSNs) [14]. Because of the close relationship between neuronal activity, arterial supply and oxygen consumption, ASL can provide complementary information to Blood Oxygen Level-Dependent (BOLD) signal [16]. Therefore, even ASL data can be used in order to evaluate brain functional networks at rest. Indeed, in the last decade, ASL has been employed, in addition to BOLD rsfMRI, to derive ICs representing network of brain metabolism, detecting intrinsic functional patterns of the resting brain [17,18,19]. In addition to applying ICA to time series data (like rsfMRI or ASL), the technique can be used with individual quantitative MRI maps acquired in a group of subjects. In this work we use ICA to capture spatial covariance information about blood flow across subjects, by deriving ICs from CBF maps (CBF-ICs).

In this framework, assessing if CBF spatial patterns can be robustly identified, and testing their consistency across different datasets is important to validate the use of CBF-ICs for future brain aging and disease studies. Therefore, the first aim of this study was to assess the stability and consistency of ICA-derived CBF patterns across different groups of healthy individuals (HI). Moreover, given that BOLD signal is closely associated with CBF due to the neurovascular coupling, we aimed to assess the degree of similarity between the CBF-ICs and the well-known spatial patterns of RSNs derived from BOLD-rsfMRI. Finally, as CBF relies on the arterial supply, the similarity between CBF-ICs and vascular territories (VT) was tested, to evaluate if the identified CBF spatial patterns mirrored the anatomical vascular distribution.

## 2. Materials and Methods

### 2.1. MRI Acquisition

Data from ninety-six HI without any neurological or neuropsychiatric disorder were included in this study. The study was performed in accordance with the principles of the Helsinki Declaration and it was approved by the Fondazione don Gnocchi’s ethical committee on July 24th 2015 with the following project identification code: 3_1/7/2015. Written informed consent was obtained from all participants.

All the enrolled subjects underwent a magnetic resonance imaging (MRI) acquisition on a 1.5 T Siemens Avanto scanner equipped with a 12-channel head coil. The complete MRI protocol included: (1) a 3D high-resolution magnetization-prepared rapid gradient echo (MPRAGE) T1-weighted image (repetition time (TR) = 1900 ms, echo time (TE) = 3.3 ms, inversion time (TI) = 1100 ms, matrix size = 192 × 256 × 176, resolution = 1 mm^3^ isotropic); (2) a 3D gradient and spin echo (GRASE) multi-delay pseudo-continuous arterial spin labeling (pCASL) with background suppression sequence (TR/TE = 3500/22.58 ms, labeling duration = 1500 ms, 5 post-labeling delays (PLD) = 700/1200/1700/2200/2700 ms, 12 pairs of tag/control volumes, matrix size = 64 × 64 × 32, resolution = 3.5 × 3.5 × 5 mm^3^ distance between the center of imaging slices and labeling plane = 90 mm; 3 M0 images acquired with TR = 5000 ms); (3) double echo GRE fieldmap (TR = 528 ms, TE = 4.76/9.52 ms, matrix size = 100 × 100 × 42, resolution = 3.2 × 3.2 × 3.3 mm^3^); (4) a multi-echo resting state fMRI (ME-rsfMRI) sequence (TR = 2570 ms, TE = 15/34/54 ms, matrix size = 64 × 64 × 31, resolution = 3.75 × 3.75 × 4.5 mm^3^, 200 volumes). Sixty-seven out of ninety-six subjects were scanned with the complete MRI protocol. Twenty-five out of ninety-six subjects were scanned with a reduced protocol including the MPRAGE (1) and pCASL (2) sequences. Four out of ninety-six subjects were scanned with a reduced protocol including the MPRAGE (1), double echo GRE fieldmap (3), and multi-echo resting state fMRI (4) sequences.

### 2.2. MRI Preprocessing

The MPRAGE volumes were skull stripped using the brain extraction toolbox (BET) [20] and then normalized to the Montreal Neurological Institute (MNI) standard space, by means of Advanced Normalization Tools (ANTs [21]).

The tag and control volumes of the pCASL acquisition were realigned with ANTs [21]. The estimation of the CBF maps was performed by running oxford_asl tool [22] with the following parameters: T1 of brain tissue = 1.2 s, T1 of blood = 1.36 s, tagging efficiency = 0.8 accordingly to Wang et al. and Laganà et al. previous reports [23,24]. CBF maps were calibrated according to cerebrospinal fluid magnetization with asl_calib tool [22]. Calibrated CBF maps were linearly registered to MPRAGE, and then non-linearly registered to MNI standard space with ANTs. Finally, the obtained maps were spatially smoothed with a Gaussian kernel (σ = 2.5).

The double-echo magnitude and phase field maps, mapping the field inhomogeneities, were preprocessed with FUGUE toolbox [25] in order to obtain a correctly calibrated field map in units of rad/s. This map was subsequently used to correct rsfMRI data for distortions. The acquired functional data were checked for movements and retained for further analysis only if the amount of relative head motion, as assessed with FSL FEAT [26], was below 0.5 mm. The ME-rsfMRI sequence was preprocessed with the ME-ICA algorithm described in the study by Kundu et al. [27]. The first 10 volumes (out of 200) were discarded in order to account for the magnetization stabilization. After a standard preprocessing comprising motion correction and realignment, the three echoes were combined to obtain an optimal combination volume which underwent a subsequent denoising step. The latter is based on the estimation of ICs and their classification, either in signal or noise, basing on TE dependencies. The denoised volume was then linearly aligned with the subjects’ MPRAGE with Boundary-Based registration (BBR) [28] and corrected for the distortion basing on the field maps. The functional data were then aligned to MNI standard space. Finally, the normalized volumes were spatially smoothed with a Gaussian kernel (σ = 2.5).

### 2.3. Consistency

In order to perform a consistency analysis, the ASL dataset (92 HI) was split into subsamples according to three different criteria. In particular, we evaluated the consistency between (1) two randomly split age and sex-matched subgroups (G1, G2) with equal sample size, (2) elderly vs. young subjects (performing the split with respect to the median age), and (3) male vs. female subjects. Spatial ICA was performed with FSL MELODIC toolbox [14] on the CBF maps of each subsample. The ICA dimensionality (d) was set to 15. The stability of the CBF-ICs derived from the different subsamples was assessed by means of ICASSO toolbox [29], running on Matlab (R2013a, Mathworks), which performs iterative components estimation by slightly changing the initial condition to evaluate the mutual similarity of components clustering in signal space. ICs are then classified according to a quality index (Iq) which measures the compactness and isolation of a cluster. Each estimated component was considered reliable and retained for further analysis if the provided Iq was equal or greater than 0.8.

The consistency of CBF-ICs across the subsamples (i.e., G1, G2, elderly, young, females, and males) was assessed visually by an experienced operator. In particular, the anatomical localization of similar clusters in the GM was identified. In addition, the degree of spatial overlap between the CBF-ICs of the different subsamples was evaluated by means of the Dice Similarity Coefficient (DSC) [30]:

$$DSC_{A,B}=\;\frac{2*\left(A\cap B\right)}{A+B}$$

where *A* and *B* represent the compared components, while A∩B represents the number of common voxels between them. The DSC was classified as follows; (1) slight: between 0 and 0.20; (2) fair: between 0.21 and 0.40; (3) moderate: between 0.41 and 0.60; (4) substantial: between 0.61 and 0.80, and (5) almost perfect agreement: between 0.81 and 1 [31,32].

### 2.4. Similarity

To perform the similarity analyses of CBF ICs with both RSNs and VT, the whole ASL dataset was considered (92 HI). Spatial ICA was performed with FSL MELODIC toolbox [14] on the normalized CBF maps, setting d = 20. The stability of the CBF-ICs estimated from the whole ASL dataset was assessed using Icasso.

RSNs were derived considering the whole rsfMRI dataset (71 HI). Similarly, to CBF ICA, rsfMRI components estimation was performed with FSL MELODIC toolbox [14], setting d = 20. The derived components were visually inspected to identify the RSNs [33].

The similarity between CBF-ICs and the RSNs was first assessed visually by an experienced operator. Once the CBF-ICs visually similar to each RSN were identified, the DSC index was used to quantify their overlap.

Finally, the similarity between CBF-ICs and VT was evaluated. We used a VT atlas [34] which identifies the perfusion territories, according to the main anatomical branching of the cerebral arteries, namely left and right anterior, middle, and posterior cerebral arteries (ACA, MCA, and PCA, respectively). The similarity between CBF-ICs and VT was first qualitatively assessed by visual inspection, and then quantified in terms of spatial overlap with DSC.

## 3. Results

### 3.1. Consistency

The demographics of the subsamples included in the consistency analyses (random split, age split, and sex split) are presented in Table 1.

The CBF-ICs, estimated from the different subsamples, exhibited an Iq close to 1 and were retained for further analyses.

**Figure 1:**
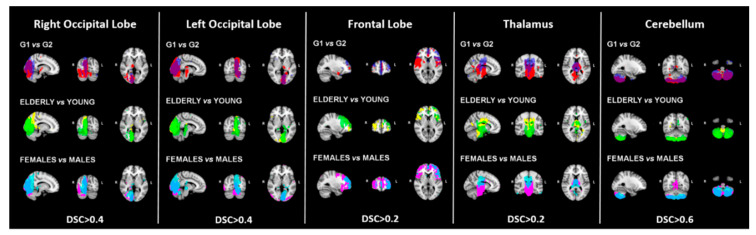
The overlaps of the most consistent CBF-ICs derived from the different splitting are displayed in the figure. The first row shows the comparison between age and sex-matched groups G1 and G2 (G1 is displayed in red while G2 in blue), the second compares groups divided according to the median age (elderly in yellow and young in green), and the third row is the overlap between males vs. females CBF-ICs (light-blue and pink respectively). Legend: G1 = group 1, G2 = group 2, DSC = Dice Similarity Coefficient.

The CBF patterns were consistently represented across the considered sub-samples, independently of age and sex. Specifically, the following CBF-ICs were the most consistent with DSC ranging from fair to moderate overlap: right occipital cortex (DSC > 0.4), left occipital cortex (DSC > 0.4), frontal cortex (DSC > 0.2), thalamus (DSC > 0.2), and cerebellum (DSC > 0.6), see Figure 1.

### 3.2. Similarity

The whole ASL dataset included in similarity analyses (92 HI) was composed of 47 males, and was characterized by a mean age of 42.8 ± 17.5 years. The whole rsfMRI dataset (71 HI) was composed of 37 males, and was characterized by a mean age of 45.3 ± 17.4 years.

All the 20 CBF-ICs, estimated from the whole ASL dataset, exhibited an Iq > 0.85 and were retained for the similarity analyses.

Fourteen out of 20 rsfMRI components were classified as RSNs.

**Figure 2:**
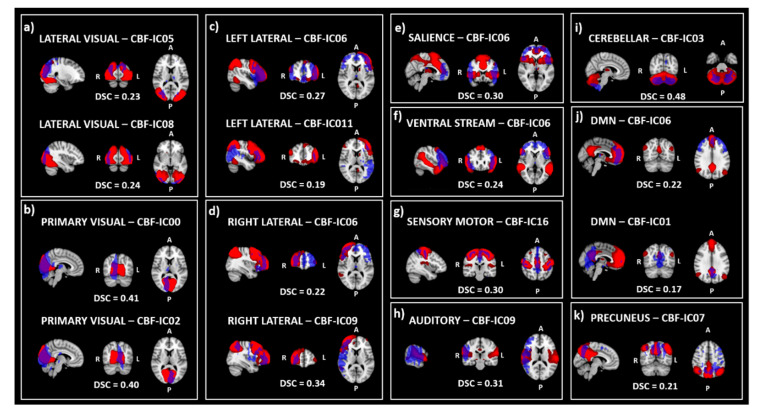
The figure shows the comparison between CBF-ICs (in blue) and the resting state network (RSN) (in red) derived from the whole ASL and rsfMRI sample, respectively. Specifically, the overlap between CBF-ICs and lateral visual network (**a**), primary visual network (**b**), left and right lateral networks (**c**,**d**), salience network (**e**), ventral network (**f**), sensory-motor network (**g**), auditory network (**h**), cerebellum (**i**), default mode network (**j**), and precuneus network (**k**) are reported. Legend: RSN = Resting state network; CBF-ICs = independent components derived from CBF maps; DSC = Dice Similarity Coefficient; DMN = Default mode network.

Fair (DSC ≥ 0.20) to moderate (DSC ≥ 0.40) agreement was found between 8 CBF-ICs and RSNs (Figure 2). Specifically, CBF-IC05 and CBF-IC08 were lateralized in right and left occipital components overlapping with fair agreement (DSC = 0.23 and DSC = 0.24, respectively) with the lateral visual network (Figure 2a). A pair of right and left occipital components, CBF-IC00 and CBF-IC02, overlapped with a moderate agreement (DSC equal to 0.41 and 0.40, respectively) with the primary visual network (Figure 2b). CBF-IC06, resembling the bilateral frontoparietal RSN, overlapped both with left frontoparietal (DSC = 0.27) and right frontoparietal (DSC = 0.22) RSNs mainly in the frontal region (Figure 2c,d); however, the right lateral RSN showed a higher agreement with CBF-IC09 (DSC = 0.34) extending more posteriorly (Figure 2, panel d). CBF-IC06 showed a fair (DSC = 0.30) overlap also when compared with the salience network, and with the ventral stream network (DSC = 0.24) (Figure 2e,f, respectively). CBF-IC16 showed fair agreement with the sensory-motor network (DSC = 0.30) (Figure 2, panel g); a fair overlap resulted from the comparison between CBF-IC09 and the auditory RSN (DSC = 0.31) (Figure 2h). A moderate overlap resulted between CBF-IC03 and the cerebellar RSN (DSC = 0.48) (Figure i). Finally, the frontal and precuneus nodes of the default mode network were separately detected and overlapped with CBF-IC06/CBF-IC01 (DSC = 0.22 and DSC = 0.17, respectively) (Figure 2j); the precuneus node was also separately detected both for CBF (CBF-IC07) and rsfMRI derived components and their agreement showed a DSC = 0.21 (Figure 2k).

**Figure 3:**
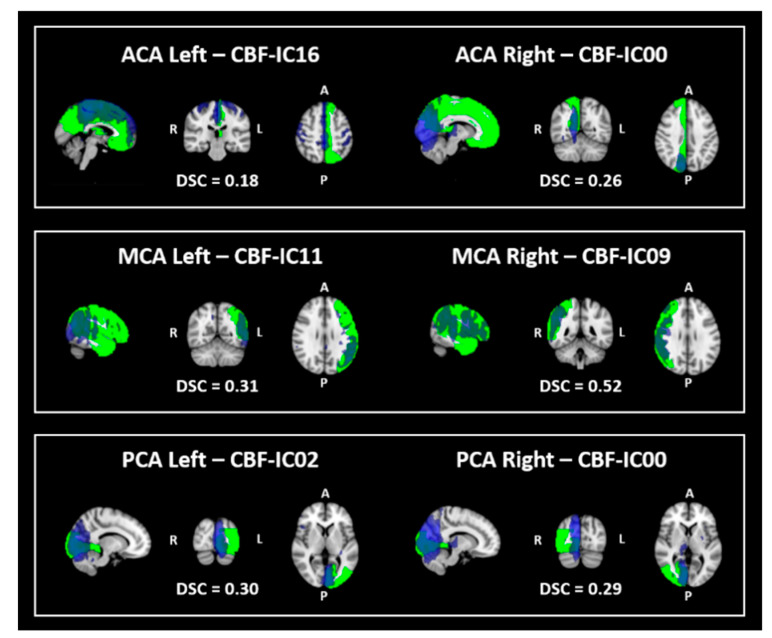
The figure shows the comparison between CBF-ICs (in blue) and the VT atlas (in green). Specifically, comparison between CBF-ICs and the left and right ACA are reported in the top panel, the middle panel shows the comparison between CBF-ICs and left and right MCA, the bottom panel shows the comparison between CBF-ICs and left and right PCA. Legend: CBF-ICs = independent components derived from CBF maps; VT = vascular territories, ACA = anterior cerebral artery, MCA = middle cerebral artery; PCA = posterior cerebral artery; DSC = Dice Similarity Coefficient.

The overlap between the CBF-ICs and the VTs is shown in Figure 3.

CBF-IC00 and CBF-IC16 showed fair and slight overlap with right (DSC = 0.26) and left ACA (DSC = 0.18), respectively. A fair to moderate overlap was found for the MCA between CBF-IC06 and left MCA (DSC = 0.31) and between CBF-IC09 and right MCA (DSC = 0.52), respectively. Finally, CBF-IC00 and CBF-IC02 overlapped, with fair confidence, with right (DSC = 0.29) and left (DSC = 0.30) PCA, respectively.

## 4. Discussion

The present study validated the consistency of the CBF-derived components in a group of HI split in subgroups, and explored CBF-ICs similarity with RSNs and VT. Some CBF-ICs were robustly identified across HI subgroups and partially mirrored RSNs and VTs, supporting the idea that CBF-ICs may capture both functional and vascular network information.

Interestingly, all the CBF-ICs estimated in this study (i.e., both in the whole sample and in the different subsample’s analyses) proved to be very stable (Iq ≥ 0.85). One of the main limitations of ICA is its stochastic nature, meaning that different runs of the algorithm can lead to different data decomposition. However, as all the detected CBF-ICs in this study corresponded to clusters of voxels that were well separated from the rest of the estimate, their reliability was confirmed, excluding statistical errors in their estimation [29] and justifying their inclusion in further analyses.

Five CBF-ICs (namely, frontal and bilateral occipital cortices, cerebellum and thalamus) were consistently identified among the six sub-groups derived from the three splits of our HI group. This result suggests that spatial patterns of perfusion covariance in the left and right occipital cortex, in the frontal cortex, thalamus, and cerebellum can be very robustly identified regardless of age and sex differences in HI. Another evidence of the stability of these components is the fact that the same CBF-ICs identified among different subsamples in the consistency analysis, were preserved in the estimate from the whole ASL dataset. Although we acknowledge that not all the estimated CBF-ICs were consistent, we believe that the five CBF-ICs that were robustly identified across groups are a remarkable result for future investigation of CBF-ICs in pathological conditions. Indeed, all the brain regions identified as part of the consistent CBF-ICs are important functional hubs, and changes in these areas were reported across the lifespan and in some neurologic and neurodegenerative diseases. The occipital lobe is the main hub of the visual pathway, and it plays an important role in the processing of visual information, that is impaired in some neurodegenerative diseases. For instance, occipital hypoperfusion was observed in Alzheimer disease (AD) [35] and Parkinson’s disease (PD) patients [16,36]. The frontal lobe has a well-established role in executive functions, and perfusion changes in the frontal cortex were reported in frontotemporal dementia, in AD [37], and in association with PD clinical severity [38]. The thalamus is a relay station between cortical and subcortical regions, and it plays major roles in cortical activation, by delivering sensory information to high-level cortical centers that influence cognition [39]. Thalamic alterations were extensively reported in multiple sclerosis (MS), and recently, even perfusion alterations in the thalamus were shown to be associated with disability [40] and cognitive performance [41] in MS. The cerebellum is involved in several functions related to movement and coordination, but also plays a role even in cognition. Cerebellar hypoperfusion was reported in a patient with spells of imbalance [42], and, more recently, it was observed during migraine attack [43]. The aforementioned perfusion alterations associated with varied neurological and neurodegenerative diseases are just few examples that suggest that investigating the five consistent CBF-ICs may be interesting. The spatial perfusion patterns that are robustly identified in HI might be investigated in pathological conditions in order to study their eventual change. CBF patterns may also be studied in association with rehabilitation treatments, for example, to investigate if the treatment induces changes in the CBF-ICs and/or if responders and non-responders present with different CBF patterns at baseline. Finally, as variations in perfusion were observed in childhood, adolescence, and late life [44], assessing the evolution of consistent CBF-ICs throughout the lifespan may help in the understanding the influence of aging on the brain.

The consistency of frontal and occipital cortices, cerebellum and thalamus CBF-ICs across sub-samples of HI further highlights that the regions stably identified in the same IC represent areas of spatial perfusion covariance. CBF spatial covariance across subjects may be either because homotopic areas receive blood supply from the same main arterial branch or as a result of being part of the same functional network. Indeed, regions that present with synchronous neural activity have similar metabolic activity and, thus, a coordinated demand for blood supply. CBF ICA does not allow distinguishing which is the main driver of CBF covariance between the two. However, basing on the results obtained in our similarity analysis, we hypothesize that both factors are involved.

In this study several CBF-ICs resembling RSNs were identified. Specifically, we observed a correspondence between CBF-ICs and occipital/visual RSNs, left and right lateral RSNs, sensory-motor RSN, salience RSN, ventral RSN, auditory RSN and the DMN (split in its constituent nodes). These findings are in line with previous studies comparing ASL-derived ICs and BOLD-derived RSNs [17,18,19]. In the studies by Liang et al. and Dai et al. [17,19] the overlap was not quantified, while Jann and colleagues [18] reported a moderate to substantial level of spatial overlap measured with DSC between ASL-ICs and RSNs. The discrepancies between our ranges of DSC and the ones reported by the literature (fair-moderate vs. moderate-substantial, respectively) are probably because CBF-ICs were extracted from CBF maps in the current study (i.e., representing spatial CBF covariance), while, in the previous study, ICs were extracted from ASL data [18] (i.e., representing both spatial and temporal correlation of brain perfusion). Although RSNs and CBF-ICs provide complementary information (e.g., synchronous brain activity and spatial distribution of blood flow differences respectively), they showed a certain degree of overlap as we expected, as they are both hemodynamically driven techniques. Interestingly, some CBF-ICs identified in our experiment were lateralized, i.e., split in left and right brain hemispheres. This aspect affected the DSC between CBF-ICs and functional RSNs, sensibly reducing it if compared to the overlap that would have been obtained when combining left and right CBF-ICs together. A clear example of this is the occipital CBF-ICs. The occipital cortex is specifically involved in visual perception. Brain activity in the occipital cortex was extensively reported in fMRI studies basing on visual stimuli [45,46], and even rsfMRI studies robustly derived visual RSN in the occipital pole [33]. The lateralization of occipital CBF-ICs reported in the current study suggests that the CBF covariance patterns depend not just on the segregation of brain regions in functional network, but also on the anatomical lateralization of its blood supply distribution (left and right PCA for the occipital cortex). Given that the CBF-derived components are capable of mirroring both the vascular anatomical segregation and the pattern of functional activity at rest, CBF ICA may not be used to selectively extract VT. In fact, fair to moderate DSC values between CBF-ICs and VT were found. Specific acquisition strategies (e.g., the use of separate labeling coils or selective inversion of spatially confined areas) are more suitable to precisely localize and distinguish VT [34,47].

The main limitation of the study is that not all the subjects included in the study were scanned with the complete MRI protocol. Nevertheless, the group of subjects for whom ASL sequence was acquired is relatively large (92 HI), and this allowed to perform consistency analysis on sub-samples of reasonable size, despite the splitting. Furthermore, all MRI data were acquired on a 1.5 T MRI scanner. Although we acknowledge that higher signal-to-noise ratio could be obtained with 3 T scanners, a multi-delay pCASL sequence with 3D GRASE readout was used to help ensure good data quality.

In conclusion, the ICA performed on CBF maps yielded to the estimation of five robust and consistent ICs in HI, corresponding to brain hub regions. The identified patterns of CBF covariance may reflect a combination of both functional and vascular networks, as perfusion spatial distribution depends both on the metabolic request and on the anatomical cerebral vascular tree that provides the blood supply. As CBF covariance patterns that are consistent in HI may be altered in pathological conditions (either due to functional deficits or vascular alterations), investigating CBF-ICs in neurological and neurodegenerative disorders could be an interesting future development.

## Figures and Tables

**Table 1:** The table shows the demographics of the sub-samples obtained from the splitting of the whole ASL dataset according to 3 different criteria (split 1: random split in age- and sex-matched subgroups; split 2: elderly vs. young; split 3: males vs. females).

	Sub-Sample 1	Sub-Sample 2	*p*-Value
**Split 1: Random**	*n* = 46	*n* = 46	
Age in yr, mean ± SD	42.4 ± 17.6	43.1 ± 17.7	0.876 ^a^
Males n (%)	24 (52%)	23 (50%)	0.835 ^b^
**Split 2: Age**	*n* = 46	*n* = 46	
Age in yr, mean ± SD	58.3 ± 10.3	27.2 ± 4.8	**<0.0001 ^a^**
Males n (%)	25 (54%)	22 (48%)	0.531 ^b^
**Split 3: Sex**	*n* = 47	*n* = 45	
Age in yr, mean ± SD	44.6 ± 17.9	40.8 ± 17.1	0.276 ^a^
Males n (%)	47 (100%)	0 (0%)	**<0.0001 ^b^**

^a^ = Mann–Whitney Independent Samples Test; ^b^ = Chi-square Test. Significant *p*-values are highlighted in bold. Legend: *n* = number, yr = years, SD = standard deviation.
